# Indoleamine 2,3-dioxygenase-1 (IDO1) expression by childhood acute myeloid leukemias inhibits T-cell production of IFN-γ and confers an unfavorable prognosis

**DOI:** 10.1186/2051-1426-1-S1-P172

**Published:** 2013-11-07

**Authors:** Sergio Rutella, Valentina Folgiero, Perla Filippini, Valentina Bertaina, Riccardo Masetti, Marco Zecca, Giuseppina Li Pira, Giovanni F Torelli, Anna Maria Testi, Alice Bertaina, Andrea Pession, Franco Locatelli

**Affiliations:** 1 Pediatric Hematology/Oncology, IRCCS Bambino Gesù Children's Hospital, Rome, Italy; 2 Pediatric Hematology/Oncology "Lalla Seràgnoli", Univeristy of Bologna, Bologna, Italy; 3 Pediatric Hematology/Oncology, IRCCS Fondazione San Matteo, Pavia, Italy; 4 Hematology, University La Sapienza, Rome, Italy; 5 Pediatrics, University of Pavia, Pavia, Italy

## 

**Figure 1:**
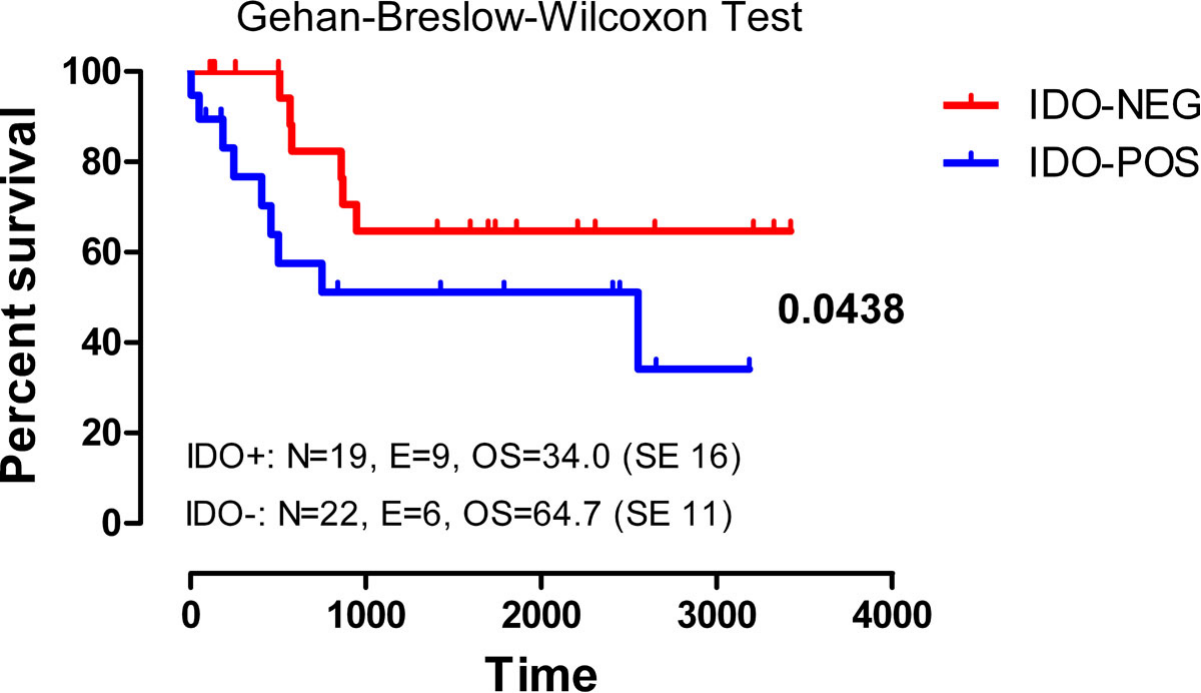
The 5-year overall survival of IDO-positive and IDO-negative patients with AML is shown.

Indoleamine 2,3-dioxygenase 1 (IDO1) degrades tryptophan into kynurenine (KYN) and other immune suppressive molecules that inhibit effector T cells and promote regulatory T-cell differentiation. We have previously shown that IDO1 mRNA and protein are detectable in blast cells from 52% of adults with newly diagnosed acute myeloid leukemia (AML). Herein, we investigated IDO1 expression and function in 41 children with AML (median age=10 years, range 1-17). In 20/41 cases, leukemia blast cells up-regulated IDO1 after in vitro challenge with IFN-γ. Of interest, microenvironmental IFN-γ was higher in IDO(pos) compared with IDO(neg) patients. In line with these results, bone marrow (BM)-resident T cells produced more IFN-γ, but not IL-4 or IL-17, compared with T cells from normal BM samples. KYN levels significantly increased in supernatants of IFN-γ-stimulated AML cells (21.0 μM/L, range 6.1-36.0) compared with unstimulated cultures (0.85 μM/L, range 0.4-1.7; p=0.0022), in parallel with tryptophan consumption (2.95 μM/L, range 1.0-37.0, after challenge with IFN-γ compared with 38.1 μM, range 18.2-50.0, in unstimulated cultures; p<0.0001). In a mixed tumor cell lymphocyte culture, AML blasts primed with IFN-γ inhibited Th1 cytokine production by allogeneic CD8+ and, to a lesser extent, CD4+ T cells, while enhancing Th2 cytokine release. The provision of D,L-1-methyl-tryptophan (1MT), an IDO inhibitor, to T-cell/AML co-cultures partially restored IFN-γ production by both CD4+ and CD8+ T cells. Furthermore, IDO-expressing AML blasts inhibited NK-cell degranulation, as measured through CD107a expression. Finally, 5-year overall survival was significantly better for IDO(neg) patients (34 months) compared with IDO(pos) ones (64.7 months; p=0.0438; Figure 1). In conclusion, IDO suppresses Th1 responses/NK activity and may portend an unfavorable prognosis in childhood AML.

